# Complete genome sequence of *Geobacillus stearothermophilus* strain CS7089 isolated from a commercial agar preparation

**DOI:** 10.1128/mra.00347-26

**Published:** 2026-05-18

**Authors:** Enea Maffei, Jessica Ransome, Carmen Saez, Martin J. Loessner, Alexander Harms

**Affiliations:** 1 Institute of Food, Nutrition and Health, D-HEST, ETH Zürich 31089 , Zürich, Switzerland; DOE Joint Genome Institute, Berkeley, California, USA

**Keywords:** thermophilic bacteria, *Geobacillus*, food microbiology

## Abstract

*Geobacillus stearothermophilus* strain CS7089 was isolated as a thermophilic contaminant from commercial agar used to produce microbiological growth media. We present the 3,555,170 bp genome of this organism and report on its basic characteristics, as well as its isolation from molten LB agar medium.

## ANNOUNCEMENT

**Fig 1:**
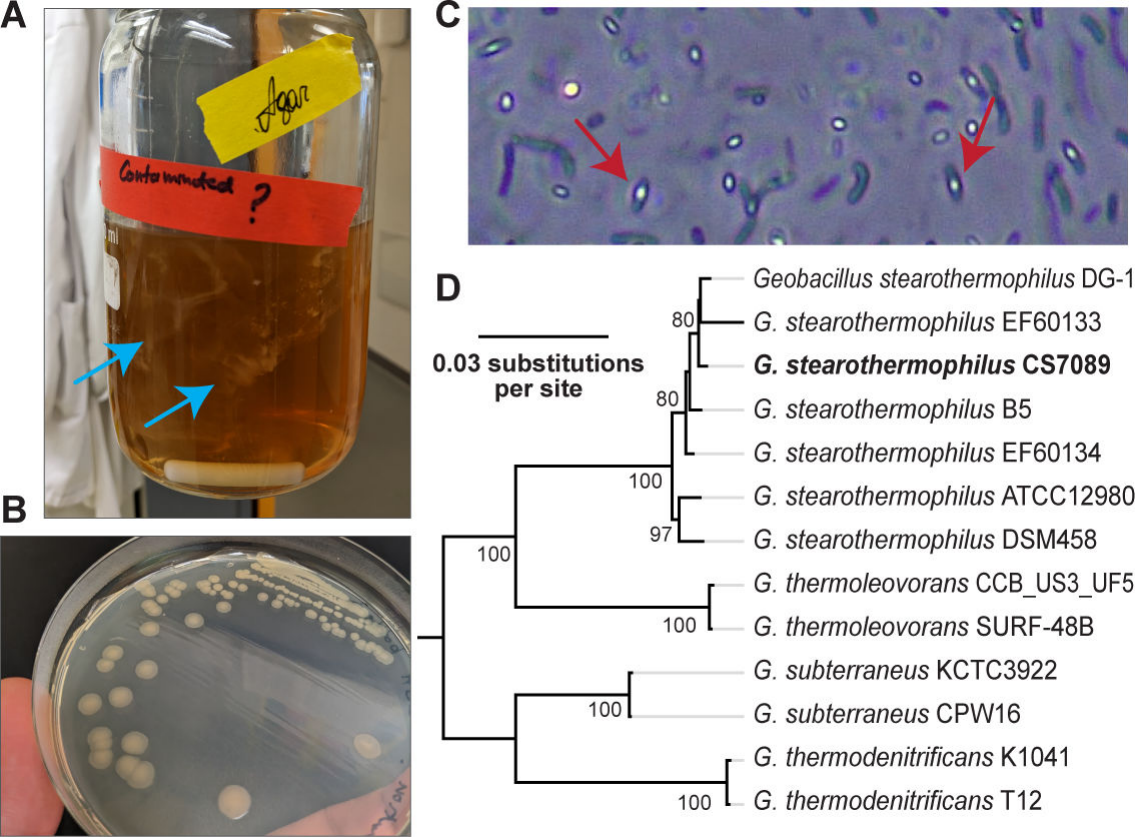
Thermophilic contamination in agar media and phylogeny of different *Geobacilli*. (**A and B**) Representative images of a contaminated LB agar bottle after storage at 60°C overnight (**A**) and colonies obtained from cultivation of the contaminant on LB agar plates at 60°C (**B**). (**C**) Brightfield microscopy of a liquid culture of a thermophilic contaminant reveals characteristic endospores (red arrows) in rod-shaped bacterial cells. (**D**) To analyze the relationship of *G. stearothermophilus* CS7089 to other *geobacillus* strains, we aligned core genes of diverse relatives (*atpD*, *dnaK*, *infB*, *rpoB*, *rpoC*) using MAFFT (v7.490) implemented in Geneious Prime 2024.0.2 with FFT-NS-1 algorithm, 200 PAM/k = 2 scoring matrix, gap open penalty 1.53, and offset value 0.123 (default settings; alignment length 22,175 nt) (12). Subsequently, we calculated a maximum likelihood phylogeny based on this alignment using PhyML (version 3.3.20180621) (13) implemented in Geneious Prime 2024.0.2 with the HKY85 substitution model and 100 boostraps. The phylogeny was midpoint-rooted between a clade formed by *G. stearothermophilus* with *G. thermoleovorans* and the other strains. Bootstrap values are shown if >70/100, and *G. stearothermophilus* CS7089 is highlighted in bold.

Solid growth media for microbiology are commonly produced by solidifying liquid media in Petri dishes with agar as gelling agent (“agar plates”) (1). For enhanced flexibility, we began storing liquefied LB agar medium (from commercial Lennox L powder) at 60°C after autoclaving. However, we noticed that hot storage of liquefied LB agar medium resulted in visible turbidity caused by microbial growth (Fig. 1A). The contaminating organism could be readily cultivated at 60°C, but not at room temperature or 37°C (Fig. 1B), indicative of a thermophilic bacterium. For identification, we cultivated a single colony in liquid LB medium at 60°C with agitation at 120 rpm. Microscopic analyses suggested a Gram-positive spore former (Fig. 1C), indicating a contamination with spores that survived our autoclaving routines. We extracted microbial DNA from 1.5 mL of contaminant culture using the GenElute Bacterial Genomic DNA Kit and transferred it to SeqVision (Vilnius, Lithuania) for sequencing. There, the genomic DNA was end-repaired with 3′ dA overhangs, and native barcodes of Oxford Nanopore kit 14 were added by T/A ligation. Subsequently, samples were purified using magnetic beads, pooled, and Nanopore native adapters were ligated (Kit 14 chemistry). DNA sequencing was performed on an R10.4.1 Nanopore flow cell with basecalling using Oxford Nanopore’s Dorado v0.9.5 (super-accuracy v5.0.0 model) and read demultiplexing with the same software. After removal of the lowest-quality 10% of reads and all shorter than 1,000 bases using Filtlong v0.3.1 (2), we obtained a total of 149,763 reads (415,672,307 bases in total) with an N50 length of 7,859 bp. Reads were assembled using Autocycler v.0.5.2 (3) in fully automated mode and the resulting single circular contig of 3,555,170 bp (mean read depth 116.9×; GC content 52.3%, BUSCO completeness 99.1% [4]) was further polished using Oxford Nanopore’s Medaka v.2.1.0. We used Bakta Web with standard settings (5) to annotate 3,509 protein-coding genes as well as 89 tRNAs, 29 rRNA genes, eleven non-coding RNAs, and the transfer-messenger RNA *ssrA*. Phylogenetic analyses identified our thermophilic contaminant as *G. stearothermophilus* (Fig. 1D), with an average nucleotide identity to type strain NCA 26 (6) (ATCC 12980) of 96.69% as determined with OrthoANI with default settings (7). Interestingly, the genomes of our strain and closely related strains DG-1 or EF60133 have sizes of ca. 3.5 Mbp, while the type strain has a smaller genome of 2.83 Mbp. Spores of *G. stearothermophilus* are highly resilient to wet heat and thus not only a frequent cause of “flat sour” spoilage of canned foods (8) but also a commonly used indicator for the sterilization efficacy of autoclaving routines (9). We observed *Geobacillus* spores in agar from different suppliers, likely because agar is commercially extracted from boiling seaweeds, which may create a suitable niche for these bacteria (10). Our laboratory therefore now applies a heat pulse (100°C for 25 min) to trigger endospore germination in agar-containing media after which autoclaving reliably eliminates all bacteria analogous to classical Tyndallization (11). This work does therefore not only present a new strain of *G. stearothermophilus* but also highlights links between basic microbiology techniques and food spoilage.

## Data Availability

The whole genome sequencing project for *Geobacillus stearothermophilus* CS7089 has been deposited in the European Nucleotide Archive (ENA) database under accession number PRJEB110097 (biosample accession SAMEA121971454, assembly accession GCA_982319185, and read accession ERR16838201), with the genome version described in this paper as the original.
